# Experimental Pulmonary Hypertension Is Associated With Neuroinflammation in the Spinal Cord

**DOI:** 10.3389/fphys.2019.01186

**Published:** 2019-09-20

**Authors:** Mylene Vaillancourt, Pamela Chia, Lejla Medzikovic, Nancy Cao, Gregoire Ruffenach, David Younessi, Soban Umar

**Affiliations:** Department of Anesthesiology and Perioperative Medicine, University of California, Los Angeles, Los Angeles, CA, United States

**Keywords:** pulmonary hypertension, Monocrotaline, neuroinflammation, spinal cord, oxidative stress, sympathetic nervous system, sympathetic activation

## Abstract

**Rationale:**

Pulmonary hypertension (PH) is a rare but fatal disease characterized by elevated pulmonary pressures and vascular remodeling, leading to right ventricular failure and death. Recently, neuroinflammation has been suggested to be involved in the sympathetic activation in experimental PH. Whether PH is associated with neuroinflammation in the spinal cord has never been investigated.

**Methods/Results:**

PH was well-established in adult male Wistar rats 3-week after pulmonary endothelial toxin Monocrotaline (MCT) injection. Using the thoracic segments of the spinal cord, we found a 5-fold increase for the glial fibrillary acidic protein (GFAP) in PH rats compared to controls (*p* < 0.05). To further determine the region of the spinal cord where GFAP was expressed, we performed immunofluorescence and found a 3 to 3.5-fold increase of GFAP marker in the gray matter, and a 2 to 3-fold increase in the white matter in the spinal cord of PH rats compared to controls. This increase was due to PH (MCT vs. Control; *p* < 0.01), and there was no difference between the dorsal versus ventral region. PH rats also had an increase in the pro-inflammatory marker chemokine (C-C motif) ligand 3 (CCL3) protein expression (∼ 3-fold) and (2.8 to 4-fold, *p* < 0.01) in the white matter. Finally, angiogenesis was increased in PH rat spinal cords assessed by the adhesion molecule CD31 expression (1.5 to 2.3-fold, *p* < 0.01).

**Conclusion:**

We report for the first time evidence for neuroinflammation in the thoracic spinal cord of pulmonary hypertensive rats. The impact of spinal cord inflammation on cardiopulmonary function in PH remains elusive.

## Introduction

Pulmonary arterial hypertension (PAH) is a rare but fatal disease characterized by elevated pulmonary vascular pressure and pulmonary arterial remodeling, leading to right ventricular failure and patient’s death. The pathogenesis of PAH is very complex and multifactorial, including inflammation and dysregulation of the autonomic nervous response (Huertas et al., 2014; Vaillancourt et al., 2017). Several studies described an activation of the sympathetic nervous system in PAH patients, which was associated with clinical worsening and poor outcome (Velez-Roa et al., 2004; Dimopoulos et al., 2009; Wensel et al., 2009; Ciarka et al., 2010; Mak et al., 2012; Witte et al., 2016). Recently, neuroinflammation has been suggested to be involved in this sympathetic activation in experimental PH (Hilzendeger et al., 2014; Sharma et al., 2018). According to Sharma et al. (2018), inflammation in the paraventricular nucleus, which contains nerves projecting directly to the right ventricle (RV), may lead to right heart hypertrophy and failure. Therefore, it may be appealing to directly target neuroinflammation response in the brain to protect against PH. However, in clinical setting, pharmacological treatments of the central nervous system can be challenging due to physical and biochemical obstacles created by the blood-brain barrier (Bhowmik et al., 2015; Pulicherla and Verma, 2015). Furthermore, studies comparing neuroinflammation following identical traumas in the brain and spinal cord concluded for a greater inflammatory response in the spinal cord than in the brain (Schnell et al., 1999; Zhang and Gensel, 2014). Whether PH is associated with neuroinflammation in the spinal cord has never been investigated.

Astrocytes are the most abundant cells in the nervous tissue and play a controversial role in neuroinflammation (Colombo and Farina, 2016). Oxidative stress induces activation of astrocytes in dose and time-dependent manner as evident by increased expression of GFAP (Daverey and Agrawal, 2016). In acute inflammation, they secrete anti-inflammatory cytokines to limit inflammation, form glia scar in injured tissue and promote neuronal survival and myelin regeneration. On the other hand, astrogliosis may lead to detrimental effects by upregulating nuclear factor kappa B (NFkB), thus promoting secretion of pro-inflammatory chemokines such as chemokine (C-C motif) ligand (CCL) 2 and CCL-3 (Choi et al., 2014; Gowrisankar and Clark, 2016). Microglia, resident immune cells in the central nervous tissue, also are key regulators of neuroinflammation. After activation by nervous tissue injury, microglia proliferate and help scavenging altered myelin during demyelination and myelin regeneration (Carlson et al., 1998; Watanabe et al., 1999). However, as for astrocytes, overactivated microglia may become harmful to the tissue by expressing several pro-inflammatory components, including CCL3 (Murphy et al., 1995; Boutej et al., 2017), TNF-α (Kuno et al., 2005; Guadagno et al., 2013), glutamate (Takaki et al., 2012), and superoxide (Chao et al., 1992; Mead et al., 2012). Pro-inflammatory mediators, in addition to activating glial cells, affect the blood-spinal cord barrier (BSCB) by activating endothelial cells and increasing the recruitment and trans-endothelial migration of inflammatory cells into the tissue (Takeshita and Ransohoff, 2012). Here we report for the first time evidences for the presence of activated astrocytes, increased production of pro-inflammatory chemokines, as well as impaired BSCB in MCT-induced PH rat model.

## Materials and Methods

Protocols received UCLA animal research committee approval. Please refer to Supplementary Materials for details on methods.

### Animal Experiment

**FIGURE 1:**
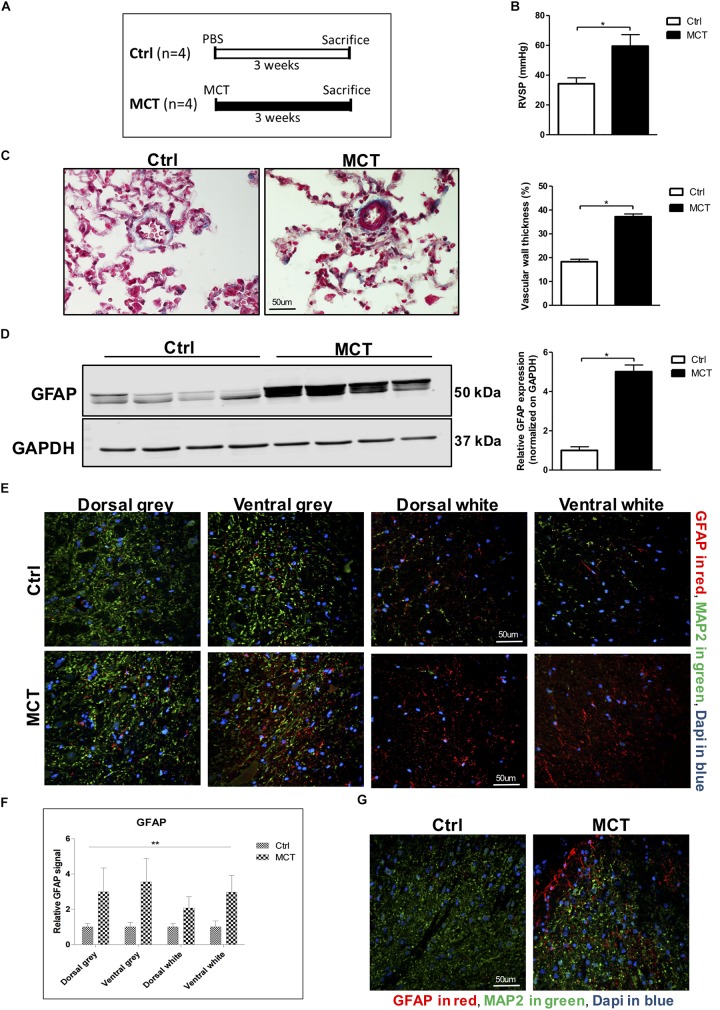
Astrogliosis is present in the spinal cord of pulmonary hypertensive rats. **(A)** Male Wistar rats received a subcutaneous injection of either MCT (60 mg/kg) or PBS for 21 days. **(B)** MCT-treated rats developed PH assessed by an increased RVSP. **(C)** Trichrome staining and quantification for pulmonary vascular remodeling. **(D)** Western blot for GFAP expression in the spinal cord. **(E)** Representative immunofluorescence for GFAP shown in red, MAP2 in green and nuclei (Dapi) in blue. **(F)** Quantification of GFAP staining in the different regions of the spinal cord. 2-way ANOVA, with factors of treatment (MCT vs. Ctrl) and region (gray vs. white matter and dorsal vs. ventral), revealed a main effect of MCT treatment, shown by (^∗∗^*p* < 0.01), with no significant main effect of the region or interaction. **(G)** Immunofluorescence showing a concentration of GFAP positive glial cells around the dorsal horn in MCT-induced PH rats. Mann-Whitney *U* test was used for comparisons between two groups. ^∗^*p* < 0.05, ^∗∗^*p* < 0.01, ^∗∗∗^*p* < 0.001.

Male Wistar rats were injected s.c. with either 60 mg/kg of crotaline (MCT, *n* = 4) or PBS (Ctrl, *n* = 4) (Figure 1A). After 21 days, animals were anesthetized and right ventricular systolic pressure (RVSP) was measured. Lungs and spinal cords were collected.

### Western Blot Analysis

Western blots were performed on thoracic segments of the spinal cord. Blots were incubated with primary antibody against GFAP.

### Immunofluorescence Staining

Thoracic spinal cord sections were stained for GFAP, CCL3, CD31, and MAP2. Images were acquired with a confocal microscope (Nikon) and analyzed.

### Enzyme-Linked Immunosorbent Assay (ELISA)

CCL3 concentration was measured on thoracic segments of the spinal cord using a rat-specific CCL3 ELISA kit according to manufacturer’s instructions.

### Statistical Analysis

Values were expressed in fold changes or mean ± SEM. Mann-Whitney *U* test was used for comparisons between two groups. 2-way ANOVA, with factors of treatment (MCT vs. Ctrl) and region (gray vs. white matter and dorsal vs. ventral), was performed when comparing the different regions of the spinal cord. Probability values <0.05 were considered statistically significant.

## Results

### Astrogliosis Is Present in the Spinal Cord of Pulmonary Hypertensive Rats

Pulmonary hypertension in MCT-treated rats was confirmed by increased RV pressure and pulmonary vascular remodeling (Figures 1B,C). To assess the presence of astrogliosis in PH, we performed a Western blot for GFAP, a marker for astrocyte activation. GFAP expression was significantly increased by 5-fold in MCT-induced PH rats compared to controls (*p* < 0.05) (Figure 1D). To further determine in which region of the spinal cord GFAP was expressed, we performed immunofluorescence of thoracic spinal cord tissue sections in combination with the microtubule-associated protein 2 (MAP2) marker to distinguish between the gray and the white matter. We found a 3 to 3.5-fold increase of GFAP marker in the gray matter, and a 2 to 3-fold increase in the white matter in the spinal cord of our PH rats compared to controls (Figures 1E,F). There was no impact of the region of the spinal cord (dorsal vs. ventral and gray vs. white matter) on this increase of GFAP. However, there was a significant effect (*p* < 0.01) of PH development (MCT vs. Control) on GFAP expression in the spinal cord tissue (Figure 1F). Interestingly, we observed an accumulation of activated astrocytes around the outer laminae of the dorsal horn (Figure 1G), which receives terminals of primary afferent and sensory neurons, in opposition to the ventral horn which contains efferent and motor neurons.

### Pro-inflammatory Chemokine CCL3 Is Increased in Spinal Neuronal Tissue From PH Rats

**FIGURE 2:**
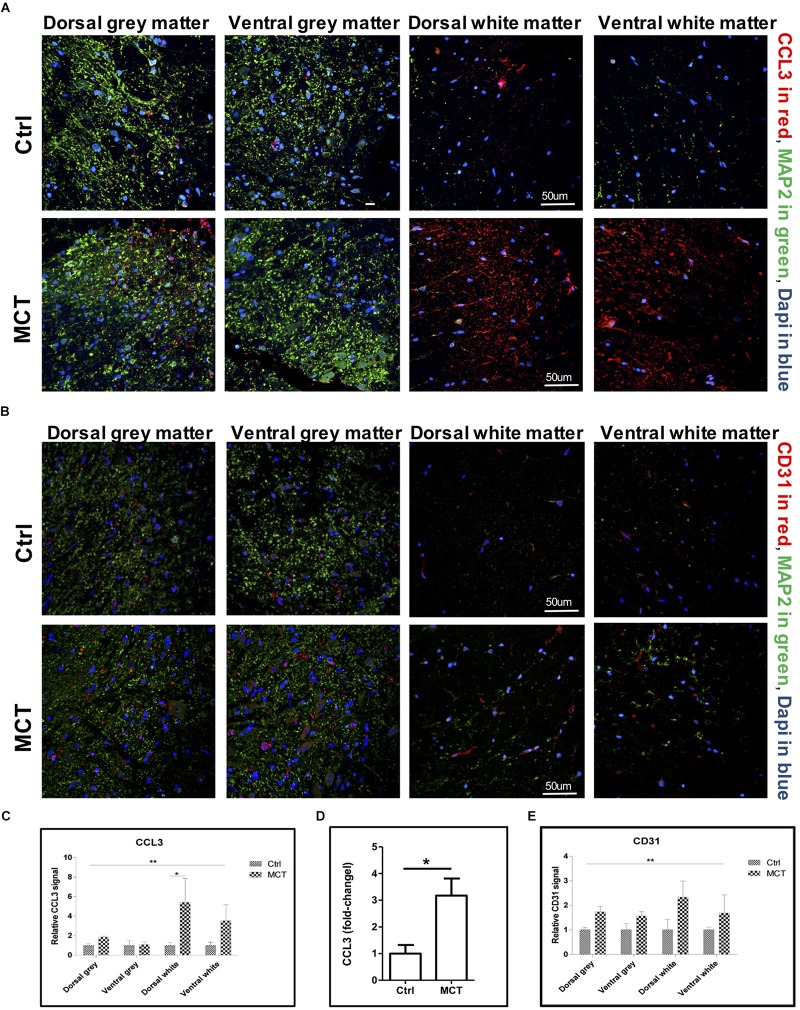
Pulmonary hypertension is associated with increased pro-inflammatory markers and angiogenesis. **(A)** Representative immunofluorescence showing CCL3 in red, MAP2 in green and nuclei (Dapi) in blue. **(B)** Representative immunofluorescence showing the adhesion molecule CD31 expression in red, MAP2 in green and nuclei (Dapi) in blue. **(C)** Quantification of CCL3 expression using a 2-way ANOVA, with factors of treatment (MCT vs. Ctrl) and region (gray vs. white matter and dorsal vs. ventral), revealing an effect of MCT treatment shown by (^∗∗^*p* < 0.01), with no significant main effect of the region or interaction except for dorsal white (^∗^*p* < 0.05). **(D)** Quantification of CCL3 expression in the thoracic section of the spinal cords using ELISA (^∗^*p* < 0.05). **(E)** Quantification of CD31 expression using a 2-way ANOVA, with factors of treatment (MCT vs. Ctrl) and region (gray vs. white matter and dorsal vs. ventral), revealing a main effect of MCT treatment shown by (^∗∗^*p* < 0.01), with no significant main effect of the region or interaction. Mann-Whitney *U* test was used for comparisons two groups.

Detrimental astroglial activation is characterized by the release of pro-inflammatory cytokines and chemokines, which may lead to other glial cell activation and leukocyte recruitment in neuronal tissue. We looked for the presence of CCL3, one of these chemokines, in the spinal cord and found a -2.5 to 4-fold increase in CCL3 staining in the white matter of PH rats compared to controls. This increase was due to the development of PH (*p* < 0.01) and the expression increased in dorsal and ventral white parts but was not different between the dorsal and the ventral gray parts of the thoracic spinal cord (Figures 2A–C). Furthermore, CCL3 expression in the thoracic section of the spinal cord was also increased ∼ 3-fold in rats treated with MCT compared to controls, as measured by ELISA (Figure 2D).

### Experimental PH Is Associated With Spinal Neurovascular Endothelial Activation

CD31 is an adhesion molecule member of the immunoglobulin superfamily highly expressed by endothelial cells. CD31, in addition of being an endothelial marker, is a key regulator of leukocyte trans-endothelial migration and angiogenesis (Woodfin et al., 2007) and was increased after spinal cord injury (Schnell et al., 1999). We then looked for possible changes in CD31 expression in the spinal cord of PH rats. By immunofluorescence, we found a 1.5 to 1.7-fold increase of the CD31 adhesion molecule in the gray matter, as well as a 1.7 to 2.3-fold increase in the white matter in the spinal cord of PH rats compared to controls (Figures 2B–E). Once again, this increase was significantly impacted by PH development (*p* < 0.01), but not dependent on the region (dorsal vs. ventral and gray vs. white matter).

## Discussion

Taken together, the presence of astrogliosis, increased production of pro-inflammatory mediators like CCL3, as well as the increased expression of the adhesion molecule CD31 are the first evidences ever-published suggesting neuroinflammation in the spinal cord of MCT-induced PH rats. Sensory neurons ending in the dorsal horn play a prominent role in the transmission and modulation of pain signals. After peripheral sensitization of sensory neurons in response to local tissue injury or inflammation, these sensory neurons release increased amounts of neurotransmitters such as substance P and calcitonin gene-related peptide (Iyengar et al., 2017). The increased release of these neurotransmitters in the dorsal horn by sensory neurons may have caused sustained astrocyte activation and neuroinflammation in the thoracic spinal cord of the PH rats in our study.

CCL3 is a pro-inflammatory chemokine poorly expressed in resting glial cells. However, activated astrocytes and microglia begin expressing CCL3 upon stimulation with TNF-α, IL1-β (Choi et al., 2014), two cytokines known to be highly increased in the plasma of PAH patients (Humbert et al., 1995; Soon et al., 2010). Recently, increased levels of chemokines such as CCL1, CCL2, and CCL3 were also found in the lungs of mice and humans with PH (Florentin et al., 2018). As for astrocytes, persistent microglial activation tends to sustain inflammation rather than resolving it. In a mouse model of retinal degeneration, microglia expressing CCL3 were shown to exacerbate inflammation and retinal degeneration, which were attenuated in CCL3 knock-out mice (Kohno et al., 2014). After stimulation with TNF-α, human brain microvascular endothelial cells increased the secretion and presentation of CCL3 on their apical surface along with a decreased expression of tight junction proteins and an upregulation of adhesion molecules (Chui and Dorovini-Zis, 2010; De Laere et al., 2017). Therefore, it is possible to extrapolate a similar role for CCL3 on the BSCB integrity during neuroinflammation.

The BSCB integrity is essential for preventing inflammatory immune cell infiltration in the tissue and its disruption precedes the post-traumatic inflammatory response in spinal cord injury (Schnell et al., 1999). Moreover, the microvascular endothelium in the spinal cord is vulnerable to demyelinating inflammatory diseases, supported by observations of increased BSCB permeability related to the severity of disease in models of multiple sclerosis (Kirk and Karlik, 2003; Roscoe et al., 2009). Increased angiogenesis was shown in demyelinated lesions of a multiple sclerosis model and was persistent with chronic inflammation, perivascular infiltration and severity of the disease (Kirk and Karlik, 2003; Roscoe et al., 2009; Holley et al., 2010). In the same manner, the increased expression of CD31 in the spinal cord of our PH rats may promote recruitment and migration of leukocytes in the spinal cord of PH rats, thus sustaining neuroinflammation.

Our study suggests the presence of neuroinflammation in the spinal cord in experimental PH. Whether this neuroinflammation has direct or indirect impact on pulmonary artery pressures or right heart deterioration still needs to be investigated. It has recently been suggested that microglial activation in the CNS was playing a role in PH development (Sharma et al., 2018). Local inhibition of microglial activation in the paraventricular nucleus of PH rats prevented sympathetic activation compared to sham-treated rats, assessed by decreased plasma level of norepinephrine and restored the autonomic balance. This was associated with improvement of pulmonary pressures, vascular remodeling, and right ventricular function (Sharma et al., 2018). The upper thoracic segments of the spinal cord contain preganglionic sympathetic neurons innervating the heart and lungs, thus controlling their autonomic activity. Hence, it is possible to think that oxidative stress and neuroinflammation in this location may also increase the sympathetic activity in the heart and the pulmonary circulation promoting PH and RV dysfunction. On the other hand, Quilez et al. (2011) showed that rats mechanically ventilated with a high tidal volume developed lung injury and inflammation, leading to neuronal activation in different regions of the brain, including the paraventricular nucleus. Finally, several studies showed that chronic heart dysfunction following acute myocardial infarction induces microglial activation and chronic neuroinflammation in the periventricular nucleus (Rana et al., 2010; Dworak et al., 2014; Du et al., 2015). Microglial activation, as well as the following sympatho-excitatory response, were inhibited by the administration of minocycline (Dworak et al., 2014). These studies highlight the interplay between the lung, heart, and brain. Therefore, it is possible to hypothesize a possible role for lung inflammation and RV dysfunction in triggering and/or sustaining the neuroinflammation seen in the thoracic spinal cord of our rats with PH.

In conclusion, we report for the first time evidence for the presence of neuroinflammation in the thoracic spinal cord of pulmonary hypertensive rats. Whether this neuroinflammation is triggered and/or sustained by lung inflammation and RV dysfunction, as well as its potential impact on cardiopulmonary function remains elusive.

## Author Contributions

MV, PC, LM, NC, GR, DY, and SU were responsible for collecting, analyzing, and interpreting the data. MV and SU wrote the manuscript. SU supervised the study.

## Conflict of Interest Statement

The authors declare that the research was conducted in the absence of any commercial or financial relationships that could be construed as a potential conflict of interest. The handling Editor declared a shared affiliation, though no other collaboration, with the authors at the time of the review.

## References

[B1] Bhowmik A. Khan R. Ghosh M. K. (2015). Blood brain barrier: a challenge for effectual therapy of brain tumors. *Biomed. Res. Int.* 2015:320941. 10.1155/2015/320941 25866775 PMC4383356

[B2] Boutej H. Rahimian R. Thammisetty S. S. Béland L.-C. Lalancette-Hébert M. Kriz J. (2017). Diverging mRNA and protein networks in activated microglia reveal SRSF3 suppresses translation of highly upregulated innate immune transcripts. *Cell Rep.* 21 3220–3233. 10.1016/j.celrep.2017.11.058 29241548

[B3] Carlson S. L. Parrish M. E. Springer J. E. Doty K. Dossett L. (1998). Acute inflammatory response in spinal cord following impact injury. *Exp. Neurol.* 151 77–88. 10.1006/exnr.1998.6785 9582256

[B4] Chao C. C. Hu S. Molitor T. W. Shaskan E. G. Peterson P. K. (1992). Activated microglia mediate neuronal cell injury via a nitric oxide mechanism. *J. Immunol.* 149 2736–2741. 1383325

[B5] Choi S. S. Lee H. J. Lim I. Satoh J. Kim S. U. (2014). Human astrocytes: secretome profiles of cytokines and chemokines. *PLoS One* 9:e92325. 10.1371/journal.pone.0092325 24691121 PMC3972155

[B6] Chui R. Dorovini-Zis K. (2010). Regulation of CCL2 and CCL3 expression in human brain endothelial cells by cytokines and lipopolysaccharide. *J. Neuroinflamm.* 7:1. 10.1186/1742-2094-7-1 20047691 PMC2819252

[B7] Ciarka A. Doan V. Velez-Roa S. Naeije R. van de Borne P. (2010). Prognostic significance of sympathetic nervous system activation in pulmonary arterial hypertension. *Am. J. Respir. Crit. Care Med.* 181 1269–1275. 10.1164/rccm.200912-1856OC 20194810

[B8] Colombo E. Farina C. (2016). Astrocytes: key regulators of neuroinflammation. *Trends Immunol.* 37 608–620. 10.1016/j.it.2016.06.006 27443914

[B9] Daverey A. Agrawal S. K. (2016). Curcumin alleviates oxidative stress and mitochondrial dysfunction in astrocytes. *Neuroscience* 333 92–103. 10.1016/j.neuroscience.2016.07.012 27423629

[B10] De Laere M. Sousa C. Meena M. Buckinx R. Timmermans J.-P. Berneman Z. (2017). Increased transendothelial transport of CCL3 Is insufficient to drive immune cell transmigration through the blood–brain barrier under inflammatory conditions in vitro. *Mediat. Inflamm.* 2017:6752756. 10.1155/2017/6752756 28626344 PMC5463143

[B11] Dimopoulos S. Anastasiou-Nana M. Katsaros F. Papazachou O. Tzanis G. Gerovasili V. (2009). Impairment of autonomic nervous system activity in patients with pulmonary arterial hypertension: a case control study. *J. Card. Fail.* 15 882–889. 10.1016/j.cardfail.2009.06.001 19944365

[B12] Du D. Jiang M. Liu M. Wang J. Xia C. Guan R. (2015). Microglial P2X7 receptor in the hypothalamic paraventricular nuclei contributes to sympathoexcitatory responses in acute myocardial infarction rat. *Neurosci. Lett.* 587 22–28. 10.1016/j.neulet.2014.12.026 25524407

[B13] Dworak M. Stebbing M. Kompa A. R. Rana I. Krum H. Badoer E. (2014). Attenuation of microglial and neuronal activation in the brain by ICV minocycline following myocardial infarction. *Auton. Neurosci.* 185 43–50. 10.1016/j.autneu.2014.03.007 24794248

[B14] Florentin J. Coppin E. Vasamsetti S. B. Zhao J. Tai Y. Y. Tang Y. (2018). Inflammatory macrophage expansion in pulmonary hypertension depends upon mobilization of blood-borne monocytes. *J. Immunol.* 200 3612–3625. 10.4049/jimmunol.1701287 29632145 PMC5940510

[B15] Gowrisankar Y. V. Clark M. A. (2016). Angiotensin II induces interleukin-6 expression in astrocytes: role of reactive oxygen species and NF-κB. *Mol. Cell Endocrinol.* 437 130–141. 10.1016/j.mce.2016.08.013 27539920

[B16] Guadagno J. Xu X. Karajgikar M. Brown A. Cregan S. P. (2013). Microglia-derived TNFα induces apoptosis in neural precursor cells via transcriptional activation of the Bcl-2 family member Puma. *Cell Death Dis.* 4:e538. 10.1038/cddis.2013.59 23492769 PMC3613837

[B17] Hilzendeger A. M. Shenoy V. Raizada M. K. Katovich M. J. (2014). Neuroinflammation in pulmonary hypertension: concept, facts, and relevance. *Curr. Hypertens. Rep.* 16:469. 10.1007/s11906-014-0469-1 25090964 PMC4167643

[B18] Holley J. E. Newcombe J. Whatmore J. L. Gutowski N. J. (2010). Increased blood vessel density and endothelial cell proliferation in multiple sclerosis cerebral white matter. *Neurosci. Lett.* 470 65–70. 10.1016/j.neulet.2009.12.059 20036712

[B19] Huertas A. Perros F. Tu L. Cohen-Kaminsky S. Montani D. Dorfmüller P. (2014). Immune dysregulation and endothelial dysfunction in pulmonary arterial hypertension: a complex interplay. *Circulation* 129 1332–1340. 10.1161/circulationaha.113.004555 24664216

[B20] Humbert M. Monti G. Brenot F. Sitbon O. Portier A. Grangeot-Keros L. (1995). Increased interleukin-1 and interleukin-6 serum concentrations in severe primary pulmonary hypertension. *Am. J. Respir. Crit. Care Med.* 151 1628–1631. 10.1164/ajrccm.151.5.7735624 7735624

[B21] Iyengar S. Ossipov M. H. Johnson K. W. (2017). The role of calcitonin gene-related peptide in peripheral and central pain mechanisms including migraine. *Pain* 158 543–559. 10.1097/j.pain.0000000000000831 28301400 PMC5359791

[B22] Kirk S. L. Karlik S. J. (2003). VEGF and vascular changes in chronic neuroinflammation. *J. Autoimmun.* 21 353–363. 10.1016/s0896-8411(03)00139-2 14624758

[B23] Kohno H. Maeda T. Perusek L. Pearlman E. Maeda A. (2014). CCL3 production by microglial cells modulates disease severity in murine models of retinal degeneration. *J. Immunol.* 192 3816–3827. 10.4049/jimmunol.1301738 24639355 PMC4123815

[B24] Kuno R. Wang J. Kawanokuchi J. Takeuchi H. Mizuno T. Suzumura A. (2005). Autocrine activation of microglia by tumor necrosis factor-α. *J. Neuroimmunol.* 162 89–96. 10.1016/j.jneuroim.2005.01.015 15833363

[B25] Mak S. Witte K. K. Al-Hesayen A. Granton J. J. Parker J. D. (2012). Cardiac sympathetic activation in patients with pulmonary arterial hypertension. *Am. J. Physiol. Regul. Integrat. Comp. Physiol.* 302 R1153–R1157. 10.1152/ajpregu.00652.2011 22422664

[B26] Mead E. L. Mosley A. Eaton S. Dobson L. Heales S. J. Pocock J. M. (2012). Microglial neurotransmitter receptors trigger superoxide production in microglia; consequences for microglial–neuronal interactions. *J. Neurochem.* 121 287–301. 10.1111/j.1471-4159.2012.07659.x 22243365

[B27] Murphy G. Jia X.-C. Song Y. Ong E. Shrivastava R. Bocchini V. (1995). Macrophage inflammatory protein 1-α mRNA expression in an immortalized microglial cell line and cortical astrocyte cultures. *J. Neurosci. Res.* 40 755–763. 10.1002/jnr.490400607 7629889

[B28] Pulicherla K. K. Verma M. K. (2015). Targeting therapeutics across the blood brain barrier (BBB), prerequisite towards thrombolytic therapy for cerebrovascular disorders—an overview and advancements. *AAPS Pharm. Sci. Tech.* 16 223–233. 10.1208/s12249-015-0287-z 25613561 PMC4370956

[B29] Quilez M. E. Fuster G. Villar J. Flores C. Martí-Sistac O. Blanch L. (2011). Injurious mechanical ventilation affects neuronal activation in ventilated rats. *Crit. Care* 15:R124. 10.1186/cc10230 21569477 PMC3218983

[B30] Rana I. Stebbing M. Kompa A. Kelly D. J. Krum H. Badoer E. (2010). Microglia activation in the hypothalamic PVN following myocardial infarction. *Brain Res.* 1326 96–104. 10.1016/j.brainres.2010.02.028 20156424

[B31] Roscoe W. A. Welsh M. E. Carter D. E. Karlik S. J. (2009). VEGF and angiogenesis in acute and chronic MOG((35-55)) peptide induced EAE. *J. Neuroimmunol.* 209 6–15. 10.1016/j.jneuroim.2009.01.009 19233483

[B32] Schnell L. Fearn S. Klassen H. Schwab M. E. Perry V. H. (1999). Acute inflammatory responses to mechanical lesions in the CNS: differences between brain and spinal cord. *Eur. J. Neurosci.* 11 3648–3658. 10.1046/j.1460-9568.1999.00792.x 10564372

[B33] Sharma R. K. Oliveira A. C. Kim S. Rigatto K. Zubcevic J. Rathinasabapathy A. (2018). Involvement of neuroinflammation in the pathogenesis of monocrotaline-induced pulmonary hypertension. *Hypertension* 71 1156–1163. 10.1161/HYPERTENSIONAHA.118.10934 29712738 PMC5945302

[B34] Soon E. Holmes A. M. Treacy C. M. Doughty N. J. Southgate L. Machado R. D. (2010). Elevated levels of inflammatory cytokines predict survival in idiopathic and familial pulmonary arterial hypertension. *Circulation* 122 920–927. 10.1161/CIRCULATIONAHA.109.933762 20713898

[B35] Takaki J. Fujimori K. Miura M. Suzuki T. Sekino Y. Sato K. (2012). L-glutamate released from activated microglia downregulates astrocytic L-glutamate transporter expression in neuroinflammation: the ‘collusion’ hypothesis for increased extracellular L-glutamate concentration in neuroinflammation. *J. Neuroinflamm.* 9:275. 10.1186/1742-2094-9-275 PMC3575281 23259598

[B36] Takeshita Y. Ransohoff R. M. (2012). Inflammatory cell trafficking across the blood-brain barrier (BBB): chemokine regulation and in vitro models. *Immunol. Rev.* 248 228–239. 10.1111/j.1600-065X.2012.01127.x 22725965 PMC3383666

[B37] Vaillancourt M. Chia P. Sarji S. Nguyen J. Hoftman N. Ruffenach G. (2017). Autonomic nervous system involvement in pulmonary arterial hypertension. *Respir. Res.* 18:201. 10.1186/s12931-017-0679-6 29202826 PMC5715548

[B38] Velez-Roa S. Ciarka A. Najem B. Vachiery J.-L. Naeije R. van de Borne P. (2004). Increased sympathetic nerve activity in pulmonary artery hypertension. *Circulation* 110 1308–1312. 10.1161/01.cir.0000140724.90898.d3 15337703

[B39] Watanabe T. Yamamoto T. Abe Y. Saito N. Kumagai T. Kayama H. (1999). Differential activation of microglia after experimental spinal cord injury. *J. Neurotrauma* 16 255–265. 10.1089/neu.1999.16.255 10195473

[B40] Wensel R. Jilek C. Dörr M. Francis D. P. Stadler H. Lange T. (2009). Impaired cardiac autonomic control relates to disease severity in pulmonary hypertension. *Eur. Respir. J.* 34 895–901. 10.1183/09031936.00145708 19443531

[B41] Witte C. Meyer-Arend J. U. M. Zur H. Genannt Andrié R. Schrickel J. W. Hammerstingl C. (2016). “Heart rate variability and arrhythmic burden in pulmonary hypertension,” in *Pulmonary Dysfunction and Disease*, ed. Pokorski M. (Cham: Springer), 9–22. 10.1007/5584_2016_18 27241509

[B42] Woodfin A. Reichel C. A. Khandoga A. Corada M. Voisin M.-B. Scheiermann C. (2007). JAM-A mediates neutrophil transmigration in a stimulus-specific manner in vivo: evidence for sequential roles for JAM-A and PECAM-1 in neutrophil transmigration. *Blood* 110 1848–1856. 10.1182/blood-2006-09-047431 17505016

[B43] Zhang B. Gensel J. C. (2014). Is neuroinflammation in the injured spinal cord different than in the brain? Examining intrinsic differences between the brain and spinal cord. *Exp. Neurol.* 258 112–120. 10.1016/j.expneurol.2014.04.007 10.1016/j.expneurol.2014.04.007 25017892

