# Supplementary material for: Experimental Pulmonary Hypertension Is Associated With Neuroinflammation in the Spinal Cord
Source: Front Physiol. 2019 Sep 20;10:1186. doi: 10.3389/fphys.2019.01186 (PMC6764190; doi:10.3389/fphys.2019.01186)
Supplement: Supplementary file 1 [file Table_1.docx]

**Experimental Pulmonary Hypertension is Associated with Neuroinflammation in the Spinal Cord**

Mylene Vaillancourt, M.S., Pamela Chia, M.D., Lejla Medzikovic, Ph.D, Nancy Cao, BA, Gregoire Ruffenach, Ph.D., David Younessi, Soban Umar, M.D., Ph.D.^*^

**Supplementary Materials and Methods**

Protocols received institutional review and committee approval. The investigation conformed to the National Institutes of Health Guide for the Care and Use of Laboratory Animals. Animals were randomly assigned to different experimental groups. Experimenters were not blinded to experimental conditions.

**Animal experiment**

Adult male Wistar rats (2-3 month old, 250–350g) were randomly distributed between the groups and were injected s.c. with either 60 mg/kg of crotaline (Sigma #C2401) (MCT, n=4) or PBS (Ctrl, n=4) (Figure 1A). Crotaline was dissolved as described in our previous publications (Umar et al., 2011; Matori et al., 2012). After 21 days, animals were anesthetized and right ventricular systolic pressure (RVSP) was measured by direct catheterization in the right ventricle (RV). Lungs and spinal cords were collected.

**Western blot analysis**

Western blots were performed using 50μg of proteins from thoracic segments of the spinal cord. Proteins were loaded on a 4-20% gradient Tris- HCl/SDS polyacrylamide gel, electro transferred to nitrocellulose paper, blocked with 5% non-fat dry milk, and incubated with primary antibody against glial fibrillary acidic protein (GFAP) (Abcam # ab53554, 1:1,000) overnight at 4^o^C. Blots were then indirectly labelled using infrared fluorophore conjugated anti-goat secondary antibody for 2 h, and visualized with the Odyssey™ Imaging System (Li-Cor).

**Immunofluorescence staining**

Thoracic sections of the spinal cord were isolated and fixed in 4% paraformaldehyde, immersed in 20% sucrose, mounted with the use of OCT compound, and sectioned at 6μm. Sections were stained with the primary antibodies against GFAP (Abcam # ab53554, 1:100), CCL3 (Abcam #ab9781, 1:20), CD31 (NovusBio #NB100-2284, 1:100), and microtubule-associated protein 2 (MAP2) (Abcam #ab5392, 1:1,000). The sections were mounted using Fluoromount G with Dapi (Invitrogen # 00-4959-52). Images were acquired with a confocal microscope (Nikon) and analyzed using Fiji- Image J software.

**Enzyme-linked Immunosorbent Assay (ELISA)**

Tissue lysates were prepared from snap-frozen thoracic spinal cord sections using RIPA lysis buffer (150 mM NaCl, 50 mM Tris pH 8, 1% NP-40, 0.5% sodium deoxycholate, and 0.1% SDS) containing protease and phosphatase inhibitor cocktails (Roche). CCL3 concentration was measured using a rat-specific CCL3 ELISA kit (Abnova, #KA2204, Taoyuan, Taiwan) according to manufacturer’s instructions.

**Statistical analysis**

Values were expressed in fold changes or mean±SEM. Mann–Whitney U test was used for comparisons between 2 groups. Two-way ANOVA, with factors of treatment (MCT vs. Ctrl) and region (grey vs. white matter and dorsal vs. ventral), was performed when comparing the different regions of the spinal cord. Probability values <0.05 were considered statistically significant.

**References**

Umar S, Nadadur RD, Li J, Maltese F, Partownavid P, van der Laarse A, Eghbali M. (2011). Intralipid prevents and rescues fatal pulmonary arterial hypertension and right ventricular failure in rats. Hypertension. *58*, 512-8.

Matori H, Umar S, Nadadur RD, Sharma S, Partow-Navid R, Afkhami M, Amjedi M, Eghbali M. (2012). Genistein, a soy phytoestrogen, reverses severe pulmonary hypertension and prevents right heart failure in rats. Hypertension. *60*, 425-30.
